# Prevalence of body dysmorphic disorder, gender differences and association with body mass index among medical students in Pakistan

**DOI:** 10.1007/s44192-025-00343-x

**Published:** 2025-12-20

**Authors:** Mohammad Abdullah Hameed, Taneer Abbas, Muhammad Muneeb Warriach, Natasha Nadeem, Ambreen Tauseef, Mohsin Ali Syed

**Affiliations:** CMH Lahore Medical College & Institute of Dentistry, Abdul Rehman Road, Sarwar Colony, Lahore, Punjab 54810 Pakistan

**Keywords:** Body dysmorphic disorder, Body dysmorphic disorder screener for DSM-5, Medical students, DSM-5, Body mass index, Gender differences.

## Abstract

**Background:**

Body dysmorphic disorder (BDD) is a mental disorder characterised by a preoccupation with some perceived defects or flaws in an individual’s physical appearance which often go unnoticed by others. The preoccupation causes marked stress and impairment of a person’s normal functioning. Limited studies have examined the prevalence based on the newer DSM-5 criteria for BDD, with very few done in Pakistan thus far. Studies on the association of BDD with BMI are limited and have yielded conflicting results.

**Objective:**

This study aimed to assess the point prevalence of BDD and gender differences, along with its association with BMI in medical students.

**Methods:**

This cross-sectional study recruited medical students in a medical university in Lahore. Systematic random sampling was employed, the participants’ BMI was measured and a standard self-report questionnaire was filled by participants. Pearson’s Chi-Square test was used for group comparisons and associations between categorical variables, while Spearman’s Rank Correlation was applied for continuous variables.

**Results:**

Of the 215 students included (109 males, 106 females), the prevalence of BDD was 4.65% (*n* = 10; 5 males and 5 females). The mean BMI was 23.07 ± 3.88 kg/m^2^, with 60.5% classified as normal weight. No significant association was found between BDD and BMI. Overall, 62.3% of students reported body dissatisfaction; males most often cited hair and fat, whereas females reported skin concerns, with significantly higher skin-related concerns among females (*p* = 0.010).

**Conclusions:**

BDD is notably prevalent among medical students, involving both genders equally, and is unaffected by BMI. The findings underscore the importance of addressing concerns related to outward appearance, suggesting a need for mental health support and early intervention in medical educational settings.

**Supplementary Information:**

The online version contains supplementary material available at 10.1007/s44192-025-00343-x.

## Introduction

Body dysmorphic disorder (BDD) is a psychiatric disorder characterised by a preoccupation with one or more imagined physical defects or flaws that may not be apparent to others, causing marked impairment in social, occupational, and other aspects of functioning, as well as clinically significant stress. The diagnostic criteria say that the appearance preoccupation is not to be better explained by an eating disorder. In addition, the criteria now also include repetitive behaviours such as mirror gazing, skin picking, assurance seeking and mental acts like comparing their appearance with that of others, in the fifth edition of the Diagnostic and Statistical Manual of Mental Disorders (DSM-5), published in 2013 [1]. Concern about appearance is seen and acknowledged in most cultures as a part of normal human behaviour. However, if these concerns exceed a certain degree and are either significantly distressing or impacting a person’s quality of life, the person may have BDD [2].

BDD should be considered a topic of mounting concern, as its incidence is increasing in young people [3]. In a recent systematic review, its measured prevalence ranged from 0.5 to 3.2% in the general and 1.3–5.8% in both the student and purely medical student population based on 3 studies conducted in Saudi Arabia, China and Pakistan, with a 0–54.3% prevalence in psychiatric cohorts, highlighting the importance of psychiatric vigilance [4]. The disorder is reported to have a prevalence of 5.3% in the general population of Pakistan [5], and 5.8% in medical students at a medical university in Karachi, Pakistan [6].

Despite its increasing prevalence and severe impacts, it often goes underdiagnosed or undiagnosed [7]. A significant portion of people with BDD may present to non-psychiatric specialties and not identify the mental condition they are suffering from. Even in psychiatric settings, diagnosis is not ideal, and patients are unlikely to disclose their appearance concerns [2, 8]. High comorbidity is also reported, with the most frequently occurring comorbidities being major depressive disorder, anxiety disorders, Obsessive Compulsive Disorder, and substance-use disorders [9]. Furthermore, resources are exhausted as BDD-afflicted patients seek cosmetic interventions, aiming to “fix” the imagined defects through physical treatment when they should be given psychiatric attention [8].

There is a scarcity of literature on the prevalence of BDD in Pakistan. To the best of our knowledge, only 3 studies in the country have investigated the prevalence of the disorder in a medical student population [6, 10, 11], with one conducted in 2017 solely on female university students [11]. Among these 3 studies, the one conducted in 2015 used a modified Body Image Disturbance Questionnaire instrument from a dermatological perspective, which may have affected reliability and produced exaggerated responses [10]. Moreover, as the 2008 study was conducted over a decade ago [6], the understanding of BDD has evolved since, as evidenced by the reclassification of the disorder under the obsessive-compulsive and related disorders in the DSM-5 [1]. Furthermore, gender differences have been analysed in a few studies, only two of which were conducted in Pakistan [6, 10].

Body Mass Index (BMI), formerly called the Quetelet index, is a measure for indicating nutritional status in adults. It is defined as a person’s weight in kilograms divided by the square of the person’s height in metres (kg/m^2^) [12]. While other psychological factors like anxiety or depression may play a role in BDD, we wanted to see whether an objective measurement like BMI could also influence the development of BDD, as it is a diagnosis of a person’s negative perception of their body image. It is worth considering whether BMI affects the likelihood of developing BDD, or whether individuals with BDD tend to cluster within certain BMI categories. We also found a dearth of studies investigating the relationship between BDD and Body Mass Index (BMI), with only 2 articles found to statistically analyse this association after our thorough review of existing literature [13, 14]. A Brazilian study revealed a positive association [13], while one done in Indonesia denied the presence of the said relationship [14]. With inconsistency in available literature, a need arises for further investigation to explore BMI as a potential contributor to BDD. In addition, no study to date has assessed the link between BDD and BMI strictly in medical students.

The aftermath of the disorder can be substantial and far-reaching, such as high lifetime suicide attempts and hospitalisation [15]. Furthermore, it is of utmost importance to ascertain whether BDD is present in medical students as it can affect their academic performance, and their increased social anxiety can negatively impact their ability to provide the required standard of patient care [2, 9]. Additionally, a high level of rejection anxiety can inhibit the student’s learning experience and professional growth [2, 9]. With BDD mostly following a chronic course, it is likely that students retain the disorder and its associated negative effects when they are well into their professional careers [6].

## Objective

This study conducted in medical students aimed to [1] calculate the prevalence of and gender differences in BDD, and [2] assess the relationship between BDD and BMI.

## Materials and methods

### Study design, setting and duration

This cross-sectional analytical study was carried out in CMH Lahore Medical College & Institute of Dentistry, a medical university in Lahore, from April 2024 to March 2025.

## Study sample, selection criteria & data collection

The sample size of 227 was calculated using Cochran’s formula (n = [Z^2^pq]/e^2^) with a 95% confidence level, 4.5% margin of error, and an assumed prevalence of 13.9% [16]. Eligible participants were medical students aged 18–25 across all five years who consented voluntarily. Those who declined consent or reported a prior diagnosis of eating disorders like bulimia nervosa or anorexia nervosa were excluded via questionnaire screening and no further data were collected from them.

We employed systematic random sampling stratified by academic year to ensure representation from all five medical years. Using the formula k = N/n (*N* = 750, *n* = 227), we rounded to a sampling interval of 3, selecting every third roll number from each academic year. A total of 250 students were then approached for the study. Participants were reached out in person using their roll numbers and invited for measurements. Height was measured with a stadiometer, weight with a weighing scale, and BMI (weight (kg) / height squared (m^2^)) was calculated. Students then completed an online questionnaire. Written informed consent was obtained, detailing the study’s purpose, voluntary participation, and confidentiality. All methods were carried out in accordance with relevant guidelines and regulations. No identifying information was collected. Ethical approval was granted by the university’s ethical review committee (Case21/ERC/CMH/LMC).

## Questionnaire

The online questionnaire had two sections: demographics (age, gender, year of study, BMI) and the Body Dysmorphic Disorder Screener for DSM-5 (BDDS-5), a self-report screener for Body Dysmorphic Disorder based on DSM-5 criteria by Van Rood et al. [17], used with the author’s permission. While the BDDS-5 is a standard tool, an additional open-ended question was included to identify the specific body feature causing concern. The questionnaire was administered in English, which is the standard language of instruction in the institution.

Individuals were considered a possible or likely BDD case if they answered ‘true’ to all three questions under criterion A, at least one question under criterion B and C, and ‘not true’ on question D. If individuals fulfilled criteria A, B, and C and their response to question D was ‘true’ with a previously diagnosed eating disorder ruled out, the individual was still classified as a possible or likely BDD case. The BDDS-5 showed high convergent validity (*r* >0.80) with other BDD measures (Body Image Concern Inventory, Yale-Brown Obsessive Compulsive Scale for BDD self-rating version, and Appearance Anxiety Inventory) and had strong internal consistency (Cronbach’s α = 0.87). It offers improved readability and binary response options. Unlike the Body Image Disturbance Questionnaire (BIDQ) developed by Cash et al., which assesses general body image disturbance [18], BDDS-5 directly measures probable BDD cases, reducing overestimation by excluding those with subclinical dissatisfaction.

While most existing questionnaires utilise the DSM-4, ours followed the DSM-5 criteria for BDD. It excludes individuals endorsing item D (“The only reason that I am dissatisfied with my appearance is that I think I am too fat (too heavy) or too skinny (too light)”) unless an eating disorder is ruled out. Van Rood et al. found that 10.8% of BDD-positive individuals were incorrectly excluded based on item D, despite not having an eating disorder. To address this, respondents were asked about prior eating disorder diagnoses. This allowed us to accurately distinguish likely BDD cases from those whose concerns primarily stemmed from eating disorders.

### Data analysis

Data was analyzed using the Statistical Package for Social Sciences (SPSS) version 27. Descriptive statistics summarized demographic characteristics and BMI distribution. Prevalence of BDD was calculated as the proportion of cases among the total sample. The 95% confidence interval (CI) was estimated using the Wilson score method. Pearson’s Chi-Square test assessed gender differences in BDDS-5 responses and foci of concern. Associations between BDD and BMI were examined using Pearson Chi-Square test (categorical BMI) and correlation analysis (continuous BMI). As BMI was non-normally distributed (Kolmogorov-Smirnov test, *n* = 215), Spearman’s Rank Correlation was applied to evaluate the association between BDD and BMI.

The internal consistency of the BDDS-5 was evaluated using Cronbach’s α, with values of ≥ 0.7 considered acceptable. All tests were two-tailed with significance set at *p* < 0.05. Tables and figures were used to present findings comprehensively. Effect sizes were reported: Hedges’ *g* for mean comparisons and Cramer’s V for Pearson’s Chi-Square test.

## Results

**Fig. 1:**
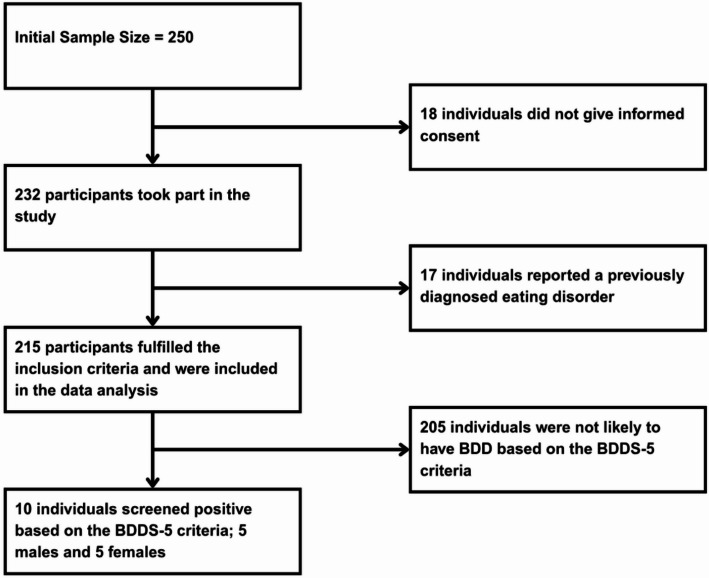
Flowchart of participant enrollment, exclusions, and BDD screening outcomes using BDDS-5

A total of 250 students were recruited for the study. After excluding 18 who did not consent to the study and 17 with prior eating disorder diagnoses, the data of 215 students were analysed. Figure 1 shows the flow of participant enrollment, exclusions, and BDD screening outcomes. The sample consisted of 109 males (50.7%) and 106 females (49.3%). The mean age was 21.04 years ± 1.58 (males: 21.18 ± 1.61, females: 20.89 ± 1.55).

The prevalence of BDD was 4.65% (10/215); 95% CI 2.55–8.35%, with an equal distribution by gender (5 males and 5 females). Cases spanned across academic years: 2 each from the first to third years, 3 from the fourth year, and 1 from the fifth year. Among males, 2 out of 5 reported body size as their concern, while 3 out of 5 females highlighted both body size and skin-related issues. Notably, 7 BDD cases answered ‘true’ to question D and denied having a prior eating disorder – these cases would have been missed had we relied upon criteria A-D, with only 3 being detected. The questionnaire demonstrated strong reliability as it had good internal consistency (Cronbach’s α = 0.84).

The mean BMI was 23.07 ± 3.88 kg/m^2^. Based on WHO criteria, 25 (11.6%) were underweight, 130 (60.5%) were normal weight, 48 (22.3%) were pre-obese, and 12 (5.6%) were obese Class 1 [13]. Table 1 displays the BMI distribution among BDD and non-BDD individuals. Among the 10 BDD cases, 2 were underweight, 3 were normal weight, 4 were pre-obese, and 1 was Obesity Class I. Although mean BMI was higher in the BDD group (24.15 ± 4.54 kg/m^2^) than in the non-BDD group (23.02 ± 3.83 kg/m^2^), Hedges’ *g* = 0.29 indicates only a small effect and the 95% CI (− 0.34 to 0.93) includes zero, so this difference is imprecisely estimated.

Pearson’s Chi-Square test found no significant association between BMI and BDD (χ^2^(df = 3) = 4.076, *p* = 0.253); Cramer’s V = 0.14, indicating a small effect size. The Fisher-Freeman-Halton exact test also indicated no significance (*p* = 0.101). BMI was non-normally distributed (Kolmogorov-Smirnov test statistic = 0.062, *p* = 0.046), so Spearman’s rank correlation was conducted to assess the relationship between BMI as a continuous variable and BDD; no significant correlation was found between BMI and BDD (ρ = 0.052, *p* = 0.449).

Table 2 compares male and female responses to BDDS-5 items. Nearly two-thirds (65.6%) of students felt others saw nothing wrong with their appearance (A3), indicating most individuals did not face judgment from their peers. About 26.0% admitted to skin-picking or changing clothes due to dissatisfaction with their outward appearance (B2), while 28.4% of students compared their appearance to others (B4). No significant difference in responses between genders was found (all *p* values > 0.05).

**Fig. 2:**
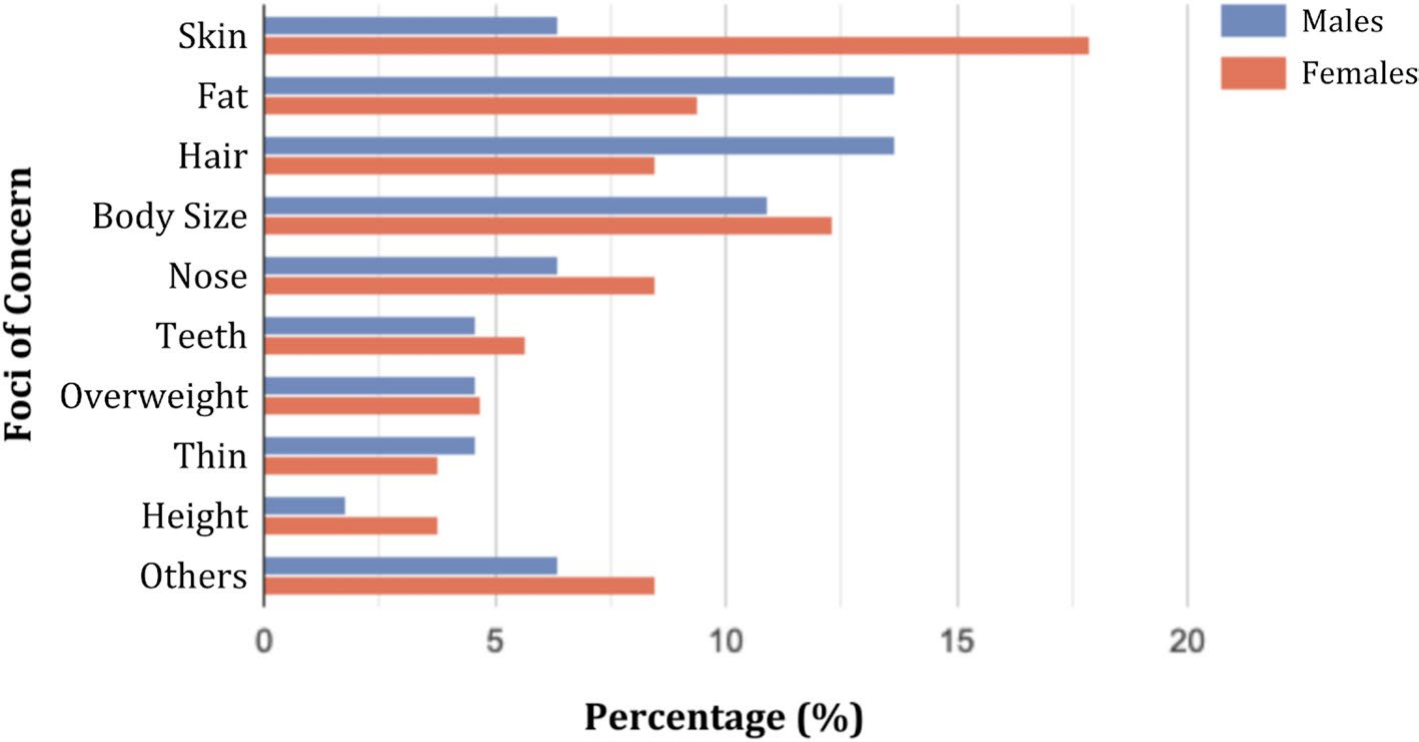
Percentages of the foci of concern among both genders

Table 3 presents the distribution of body concerns between males and females and Fig. 2 illustrates the percentages of foci of concern among both genders as a bar graph. Around 62.3% of students reported dissatisfaction with some aspect of their appearance, with similar levels between both genders (males = 62.4%, females = 62.3%). Males most commonly cited hair (e.g. thinning, receding hairline) and fat (e.g. belly fat) concerns, each reported by 15 students. Females most often were concerned by skin-related issues (e.g. acne, colour), noted by 19 students. Females were significantly more concerned about skin than males (*p* = 0.010); no other significant difference was observed between the two genders.

**Table 1:** Distribution of BMI categories among individuals with and without BDD^a^

		BMI ranges				
		Underweight	Normal weight	Pre-obesity	Obesity class I	Total
Possible or likely BDD case	No	23 (92.0%)	127 (97.7%)	44 (91.7%)	11 (91.7%)	205
	Yes	2 (8.0%)	3 (2.3%)	4 (8.3%)	1 (8.3%)	10
Total		25	130	48	12	215

^a^ Percentages represent the proportion of each BMI category

**Table 2:** Gender differences in the responses of the body dysmorphic disorder screener–5 among medical students

Questions from BDDS-5 with responses	Total (n = 215) %	Males (n = 109) %	Females (n = 106) %	*p*-value*
*A1. I think that I look strange or ugly*				
True	10.7	12.8	8.5	0.302
Not true	89.3	87.2	91.5	
*A2. I constantly think about how strange or ugly I look*				
True	9.3	8.3	10.4	0.593
Not true	90.7	91.7	89.6	
*A3. Other people do not think that there is anything wrong with my appearance, or they think that I have nothing to worry about*				
True	65.6	65.1	66.0	0.890
Not true	34.4	34.9	34.0	
*B1. I keep looking in the mirror because I am dissatisfied with my appearance, or I avoid looking in the mirror because I do not want to see that I look strange or ugly*				
True	17.7	18.3	17.0	0.793
Not true	82.3	81.7	83.0	
*B2. I constantly pick at my skin or make adjustments to my wardrobe or clothing style because I am dissatisfied with my appearance*				
True	26.0	26.6	25.5	0.850
Not true	74.0	73.4	74.5	
*B3. I keep asking other people if they think that I look strange or ugly*				
True	16.7	13.8	19.8	0.235
Not true	83.3	86.2	80.2	
*B4. I keep comparing my appearance to that of others*				
True	28.4	29.4	27.4	0.745
Not true	71.6	70.6	72.6	
*C1. I feel bad or miserable about my appearance*				
True	10.7	11.9	9.4	0.554
Not true	89.3	88.1	90.6	
*C2. I avoid doing certain things (for example, going out, dating, changing jobs, or travelling), because I am dissatisfied with my appearance*				
True	9.8	10.1	9.4	0.871
Not true	89.9	90.6	90.2	
*C3. I find it difficult to do things together with other people because I am dissatisfied about my appearance*				
True	9.3	11.0	7.5	0.382
Not true	90.7	89.0	92.5	
*C4. I have trouble focusing on my work or on a conversation because I am dissatisfied with my appearance*				
True	7.0	8.3	5.7	0.455
Not true	93.0	91.7	94.3	
*D1. The only reason that I am dissatisfied with my appearance is that I think that I am too fat (too heavy) or too skinny (too light)*				
True	19.5	21.1	17.9	0.557
Not true	80.5	40.0	40.5	

* Pearson’s Chi-Square test was used toassess significant differences in A1-D1 responses between male and female students

**Table 3:** Distribution of foci of concern reported by male and female students

Body foci of concern^a^	Total (*n* = 215)	Males (*n* = 109)	Females (*n* = 106)	*p*-value*
	Number (%)	Number (%)	Number (%)	
None	81 (37.7)	41 (37.6)	40 (37.7)	0.985
Skin-related	26 (12.1)	7 (6.4)	19 (17.9)	**0.010**
Fat	25 (11.6)	15 (13.7)	10 (9.4)	0.322
Hair	24 (11.2)	15 (13.7)	9 (8.5)	0.220
Body size	24 (11.2)	11 (10.9)	13 (12.3)	0.613
Nose	16 (7.4)	7 (6.4)	9 (8.5)	0.563
Teeth	11 (5.1)	5 (4.6)	6 (5.7)	0.721
Overweight	10 (4.7)	5 (4.6)	5 (4.7)	0.964
Thin	9 (4.2)	5 (4.6)	4 (3.8)	0.766
Height	6 (2.8)	2 (1.8)	4 (3.8)	0.388
Others^b^	16 (7.4)	7 (6.4)	9 (8.5)	—

^a^ These foci allowed multiple responses, so column percentages do not sum up to 100%

^b^ These foci included double chin, hand, face, muscle and general dissatisfaction, most of them being under 2% of the total foci of concern

* The *p*-values were obtained from Pearson’s Chi-Square Test to assess significant differences between the foci of concerns of the two genders

The bold text is for making the value prominent for the readers so they can deduce that the genderdifferences in skin-related concerns is significant

## Discussion

BDD prevalence was 4.65%, aligning with the 1.3–5.8% range reported for student populations in a 2021 review [4]. Cases were evenly split by gender. No significant association was found between BDD and BMI (χ^2^(df = 3) = 4.076, *p* = 0.253; Fisher-Freeman-Halton *p* = 0.101, Spearman’s ρ = 0.052, *p* = 0.449). Male students mainly reported concerns about body size and hair, while for females, the primary foci of concern were skin-related issues, which were significantly more common in females than in males (*p* = 0.010).

## Comparison with medical and non-medical student populations

The prevalence (4.65%) in our study is consistent with that of a similar study in Lahore, Pakistan (5%) [10]. However, higher BDD rates in medical students were reported in Karachi, Pakistan (5.8%) [6] and India (6.9%) [19], while China found a lower statistic (1.3%) [20]. These variations may be attributed to different sociocultural backgrounds and older screening tools.

Many studies have investigated BDD in non-medical students, reporting similar rates: 4.5% in Iran [21] possibly due to cultural and religious similarities, and 4.9% in the USA [22], suggesting that factors beyond cultural context – such as academic stress, body image pressure or media exposure – may play a role. In contrast, higher rates were found in Lebanon (6.4%) [23] and Germany (5.3%) [24].

Some investigations focused on gender-specific samples: 4.4% in Saudi female medical students [25], 3.3% in Malaysian male students [26], 6.1% in Pakistani female university students [11] and 4.8% in Turkish female college students [27]. However, one study in Saudi Arabia had a much higher prevalence (14.68%) [28]. Higher prevalence in females may reflect stronger societal pressures upon the appearance of women and increased likelihood of reporting body concerns.

Thus, most studies show similar trends in BDD prevalence across student populations, with exceptions like the Saudi study [28] with a prevalence nearly three times higher than ours, and the Chinese study [20] having rates nearly three times lower.

### Gender differences in BDD prevalence

Unlike many international studies that record higher prevalence in one gender, our results showed no significant gender difference. This pattern may reflect contextual factors, such as similar levels of pressure in the medical field to maintain a professional appearance, which can influence both genders similarly, overriding typical gender norms. Besides a few exceptions [6], most studies have found higher female preponderance in BDD prevalence [10, 19–24], potentially reflecting cultural factors such as societal beauty standards and media influence. Lower prevalence of BDD in males may reflect under-reporting due to stigma, while women may disclose more readily.

### Body dissatisfaction

Body image dissatisfaction is a critical concern that frequently underlies BDD. This study found that 62.3% of medical students reported dissatisfaction with some aspect of their appearance, with equal levels in males and females, demonstrating the significant burden of body image concerns in this population. This was lower than the levels seen in previous Pakistani studies. One study found dissatisfaction levels of around 70.5% in both medical and non-medical students with a female predominance [10], while another reported a dissatisfaction of 78.8% in which again, more females (88.8%) were dissatisfied than males (76.1%) [6]. In Iran, the same trend was observed, with total dissatisfied individuals being 77.7%, comprising more females (79.4%) than males (73.3%) [21]. In Turkey, this level was found to be much lower at 43.8% among a set of female college students, reflecting sociocultural differences. Body dissatisfaction is influenced by several factors, and it is found that time spent on social media has been associated with increased body dissatisfaction [29].

### Foci of concern

Among the 215 participants who had concerns regarding some part of their appearance, the most commonly reported concerns were related to the skin (12.1%), body fat (11.6%), body size (11.6%) and hair (11.2%). These findings corroborate the past trends in previous research, where skin was the most commonly reported part of the body, and hair was the third most common concern [4].

Gender analysis showed noticeable differences. Around 17.9% of females complained about skin-related issues like acne, scars or complexion, compared to just 6.4% of males, a difference which was statistically significant (*p* = 0.010). On the other hand, body fat concerns like thigh or abdominal fat were more common among males (13.7%) than females (9.4%), possibly because males tend to prefer a leaner body type. Hair-related issues showed a similar gender trend, with 13.7% of males versus 8.5% of the females mentioning it as a concern. As for body size, 12.3% of females and 10.9% of males reported concerns. These findings echo similarities with both local and international research. Aflakseir et al. also reported the top areas of body concerns for males and females to be hair and skin respectively in a sample of Iranian college students [21].

Other features like nose, teeth, thinness, and height were reported by both genders in our sample, but in very low percentages, with no major gender-based differences. However, no data was recorded for concerns related to breasts, thighs, or buttocks unlike some past studies. This may be due to Pakistan’s conservative cultural background, which has also been pointed out in earlier local research [11].

### Symptomatology

Although most students did not endorse BDD symptoms, a notable minority reported appearance-related concerns. About 10% perceived themselves as strange or ugly, and a similar proportion felt persistent distress about their looks. Behavioral features were more common, with roughly one in four reporting repetitive behaviors (26%) and nearly one in three engaging in social comparison (28%). Emotional and functional impacts, such as misery about appearance (11%) or avoidance of social activities (10%), were less frequent. No significant gender differences were observed.

Collectively, these findings indicate that while BDD symptoms are not widespread, they involve a meaningful subgroup of students, emphasizing the need for early recognition and targeted support in academic settings.

### Association of BDD with BMI

The relationship of BDD and BMI seems to have been relatively less explored in past studies, with previous literature having yielded conflicting results on whether a possible correlation between the variables exists.

For instance, a Brazilian study found a significant association between BDD and BMI, suggesting that individuals may face heightened body image concerns with weight gain [14]. In contrast, our findings align with an Indonesian study done in adolescents, which similarly reported no significant relationship between BDD and BMI [15]. Even when comparing mean BMI between groups, the difference corresponded to a small effect (Hedges’ *g* = 0.29), reinforcing that BMI differences were minor in magnitude and unlikely to be clinically meaningful. This outcome seems plausible considering that individuals with BDD experience a distorted view of themselves and are hyper aware of flaws imperceptible to others – their concerns not grounded in reality [1]. Individuals may also prioritise and obsess over other concerns such as those related to skin rather than their body weight. Thus, objective body measurements like BMI may play little to no role in giving rise to typical dysmorphic symptoms. This interpretation is consistent with the small effect size we observed (Cramer’s V = 0.14) which suggests that the association between BMI category and BDD was weak and of limited practical relevance. It should also be noted that discrepancies between studies may arise from various factors, including differences in study design and methodologies, sample characteristics, and cultural influences. Keeping in mind the multifactorial and often comorbid nature of BDD, future research may benefit from exploring additional variables – such as anxiety, depression, or exposure to social media – as potentially stronger predictors of BDD symptoms. Some studies have even indicated that BDD was linked with prolonged usage of social media applications like Instagram and Snapchat [29].

### Implications

The findings of our study have important implications for medical education and student well-being. Early screening and therapeutic strategies are essential, given the chronic course and severe consequences of BDD, including heightened suicide risk [2]. These adverse impacts may hinder the academic and professional growth of medical students, as well as their ability to provide optimal levels of patient care. Since BDD is often misdiagnosed or underdiagnosed, medical professionals should be trained in early diagnosis and management of the disorder [7].

Medical universities can also lessen stigma and encourage students to seek psychological treatment by implementing awareness campaigns, providing peer-support programs and enabling access to confidential counselling. Incorporating structured mental health and body image awareness modules into the curriculum could help normalize discussion and improve early recognition among future physicians.

Evidence-based interventions such as Cognitive Behavioral Therapy (CBT) and pharmacotherapy with selective serotonin reuptake inhibitors (SSRIs) remain the mainstay of treatment, and their integration into student mental health services may help mitigate the adverse academic and psychosocial impact of BDD [9].

### Limitations

Despite this study’s comprehensive approach, several limitations exist. First, direct measurement of height and weight may have introduced observer bias. Using multiple observers or blinding could mitigate this in future studies. Additionally, since a self-administered questionnaire was used, reporting bias is possible, including underreporting due to societal stigma around expressing appearance-related concerns. As we did not collect data on non-responders, potential differences between them and the participants could not be assessed which may introduce a selection bias.

The cross-sectional design also limits causal inference between BDD and BMI, which future longitudinal studies could address. Furthermore, the sample, being drawn from a single medical university, limits generalizability of the findings to medical students in other institutions or regions. It is also noteworthy that while our calculated sample size was 227, determined using Cochran’s formula with a reduced margin of error (4.5%), the final number of 215 was slightly lower than the intended one. This shortfall occurred despite initially recruiting 250 students. Importantly, the observed 95% confidence interval for the prevalence estimate (4.65%) remained narrow (2.55%–8.35%), suggesting this reduction had minimal impact on study precision. Nonetheless, the final sample size remains modest compared to larger epidemiological surveys, which limits the power to detect smaller effect sizes and raises the possibility of Type II errors.

Cultural influences such as media, family, and peer pressure strongly shape body image, and these perceptions differ across societies [29], so the findings of this study may not be universal. Moreover, the study did not account for psychosocial factors like anxiety, depression, or social media exposure which may act as potential confounders, influencing the BDD prevalence. Future research should consider the growing influence of digital platforms. A major limitation is that the diagnosis of BDD was based solely on a self-report screening tool without clinical interview confirmation by a psychiatrist. This may have affected the accuracy of prevalence estimates, as self-report measures are more vulnerable to under- or over- reporting of symptoms.

It is also prudent to acknowledge the limitations of the screening tool itself. The BDDS-5 was chosen for its strong psychometric properties, alignment with DSM-5 criteria and ease of use. Its high internal consistency was reflected in our study, demonstrating its reliability within a Pakistani medical student population. The BDDS-5 also has excellent convergent validity, correlating well with other BDD measures, while also being easy to administer, interpret, and score.

However, the limitations of the BDDS-5 include the lack of comparison to a gold standard tool like the Structured Clinical Interview for DSM-5 (SCID-V), leaving its diagnostic accuracy unknown. It may misclassify cases focused on weight-related dissatisfaction, though we attempted to minimize this by adding a question on eating disorders. BDDS-5’s high correlation (*r* >0.70) with Symptom Questionnaire 48 total score limits its divergent validity, though this is expected due to comorbidities in BDD patients [9]. Its factor structure was based on Exploratory Factor Analysis (EFA), but a Confirmatory Factor Analysis (CFA) in a larger population is required for stronger validity. Test-retest reliability hasn’t been assessed, so the scoring stability over a period of time is uncertain. While our study supports the use of the BDDS-5 in Pakistani medical students, broader validation is needed for generalizable results.

Nevertheless, considering the paucity of questionnaires checking for BDD in accordance with the new edition of DSM, we believed that the strengths of the used questionnaire far outweigh its weaknesses. Hence, BDDS-5 was selected for our study. Future research should incorporate clinical interviews to provide a more accurate BDD prevalence, rather than relying solely on self-reported data.

## Conclusions

This study identified a considerable prevalence of BDD among Pakistani medical students, with no gender difference in prevalence, though females reported significantly more skin-related concerns, and a lack of significant association with BMI. These findings highlight the vulnerability of medical students to body image–related issues and emphasize the need for greater awareness, support and early invention within medical educational settings. Future research across multiple institutions will be crucial to further validate these findings and guide effective preventive and therapeutic strategies.

## Supplementary Information

Below is the link to the electronic supplementary material.

Supplementary Material 1

## Data Availability

All data generated or analysed during this study are included in this published article [and its supplementary information files].

## Abbreviations

BDD: Body dysmorphic disorder
BMI: Body mass index
BDDS: Body dysmorphic disorder screener for DSM–5
DSM: Diagnostic and statistical manual of mental disorders, fifth edition
SPSS: Statistical package for the social sciences
SCID: Structured clinical interview for DSM–5
CFA: Confirmatory factor analysis
EFA: Exploratory factor analysis
WHO: World Health Organization
